# Supplementary material for: Tumor-derived exosomal miR-1247-3p induces cancer-associated fibroblast activation to foster lung metastasis of liver cancer
Source: Nat Commun. 2018 Jan 15;9:191. doi: 10.1038/s41467-017-02583-0 (PMC5768693; doi:10.1038/s41467-017-02583-0)
Supplement: Supplementary file 1 — Supplementary Information [file 41467_2017_2583_MOESM1_ESM.pdf]

# Supplementary Information

## Supplementary Figures

Supplementary Fig. 1

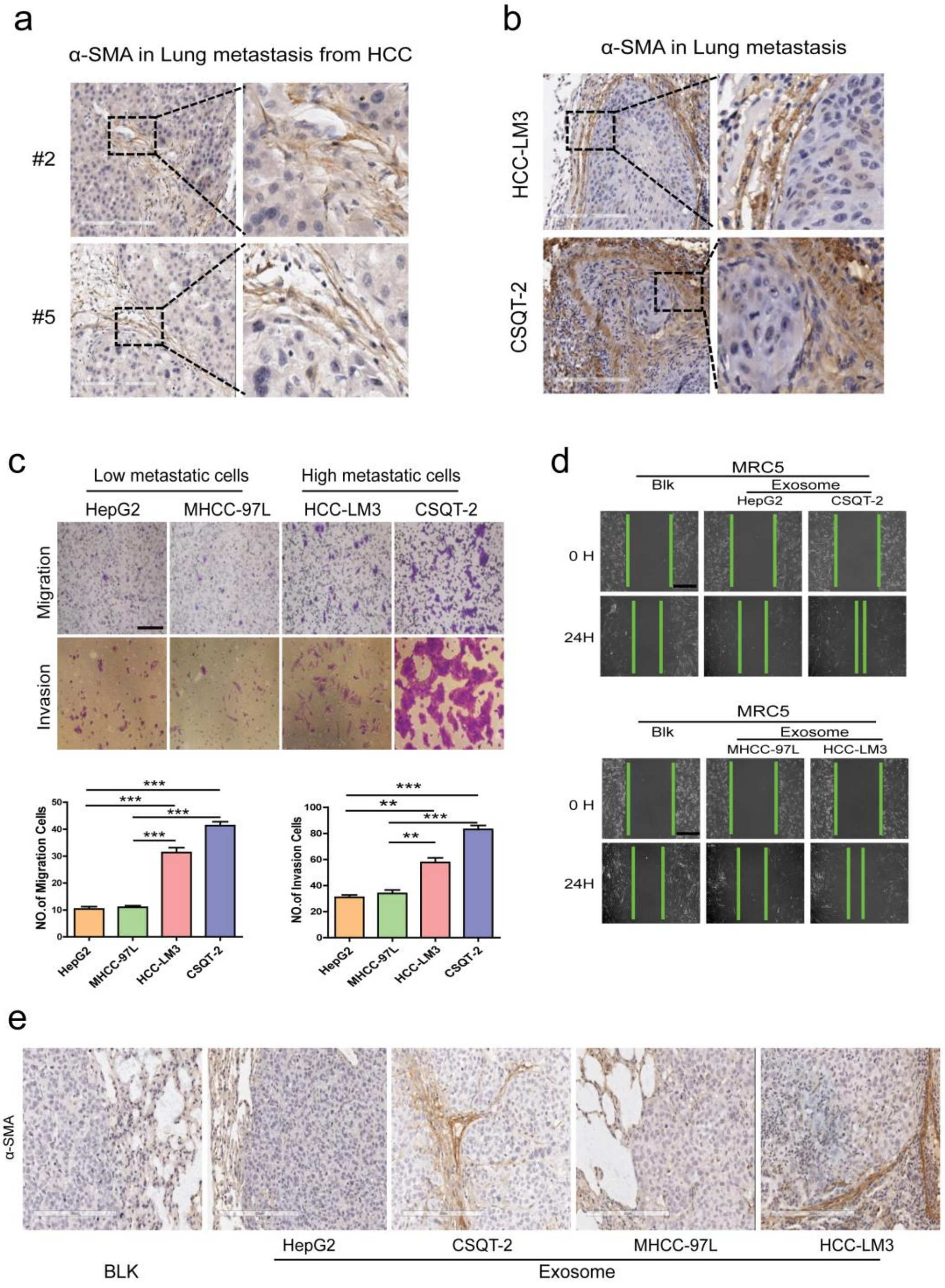


**Supplementary Fig. 1** Tumor-derived exosomes activate fibroblasts to foster lung metastasis. **a** Representative images of α-SMA staining in lung metastasis tissues from primary HCC. Scale bar, 200 μm. **b** Representative images of α-SMA staining in lung metastasis tissues from liver cancer cells in mice models. Scale bar, 200 μm. **c** Migration

and invasion assays of different liver cancer cells. Representative images were shown and cells were counted. Scale bar, 150 μm. Data are presented as mean ± s.d. Student's *t* test was used to analyze the data. (**$p<0.01$; ***$p<0.001$). **d** Wound-healing assays of MRC5 treated with equal quantities of exosomes derived from different liver cancer cells or blank control. Scale bar, 150 μm. **e** Immunohistochemistry assay of α-SMA staining in lung metastasis tumors in indicated groups. Scale bar, 200 μm.

Supplementary Fig. 2

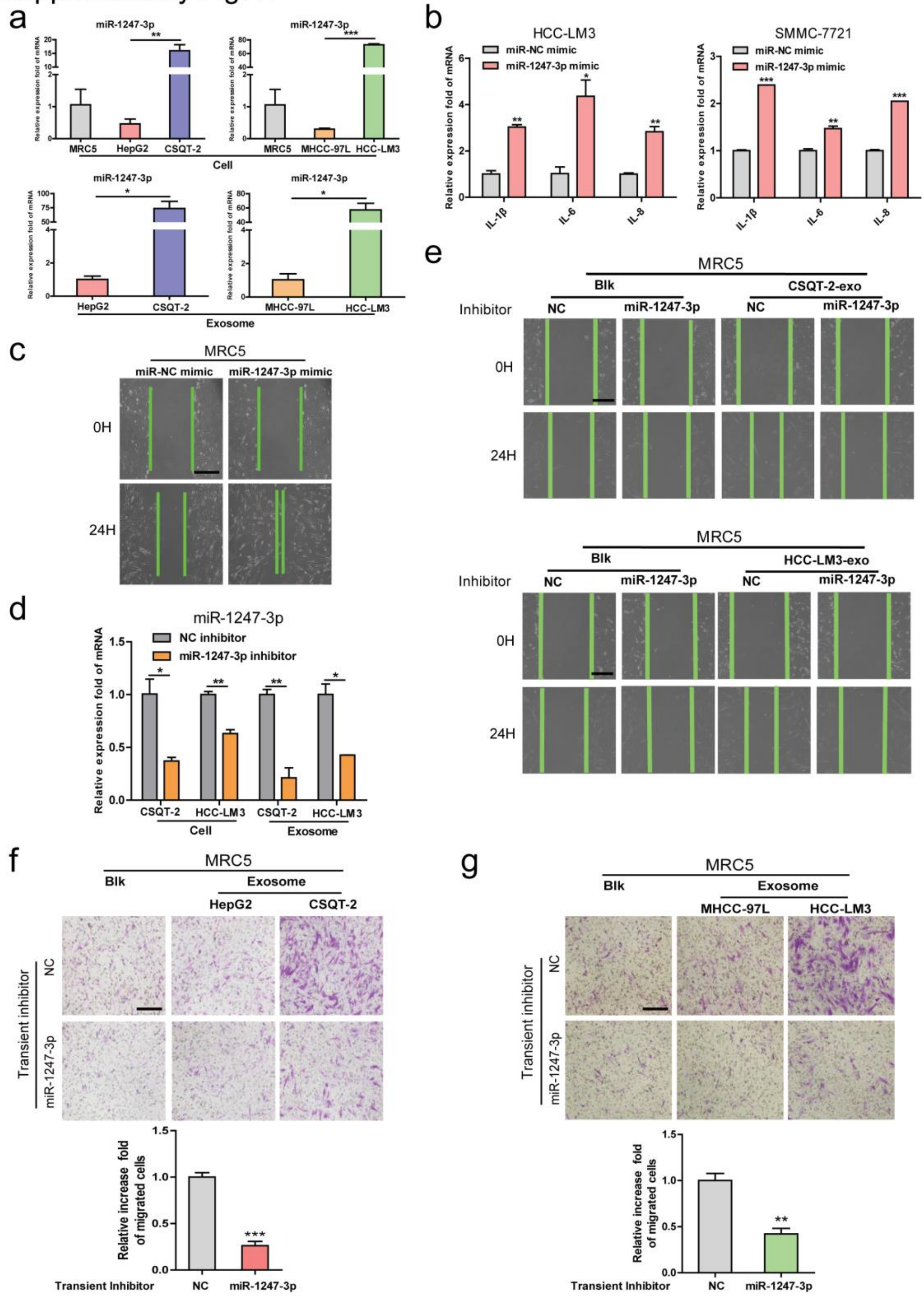


**Supplementary Fig. 2** Tumor-derived exosomal miR-1247-3p mediates fibroblasts activation. **a** qRT-PCR of miR-1247-3p in MRC5, HCC cell lines and HCC-derived exosomes. **b** qRT-PCR analysis of pro-inflammatory genes expression of HCC cells transfected with miR-1247-mimic or normal control. **c** Wound-healing assay of MRC5 transfected miR-1247-mimic or normal control. Scale bar, 150 μm. **d** qRT-PCR of miR-1247-3p in highly metastatic HCC cell lines (CSQT-2 and HCC-LM3) and

HCC-derived exosomes stably expressing miR-1247-3p inhibitor or control. **e** Wound-healing assay of MRC5 treated with indicated CM. Scale bar, 150 μm. **f**, **g** Migration assay of MRC5 treated with exosomes derived from HepG2 versus CSQT-2 or MHCC-97L versus HCC-LM3 transiently transfected with miR-1247-3p inhibitor or not. Representative images were shown and migrated cells were counted. Scale bar, 150 μm. Experiments were performed in triplicate and all data are presented as mean ± s.d. Student's *t* test was used to analyze the data. (*p<0.05; **p<0.01; ***p<0.001)

Supplementary Fig. 3

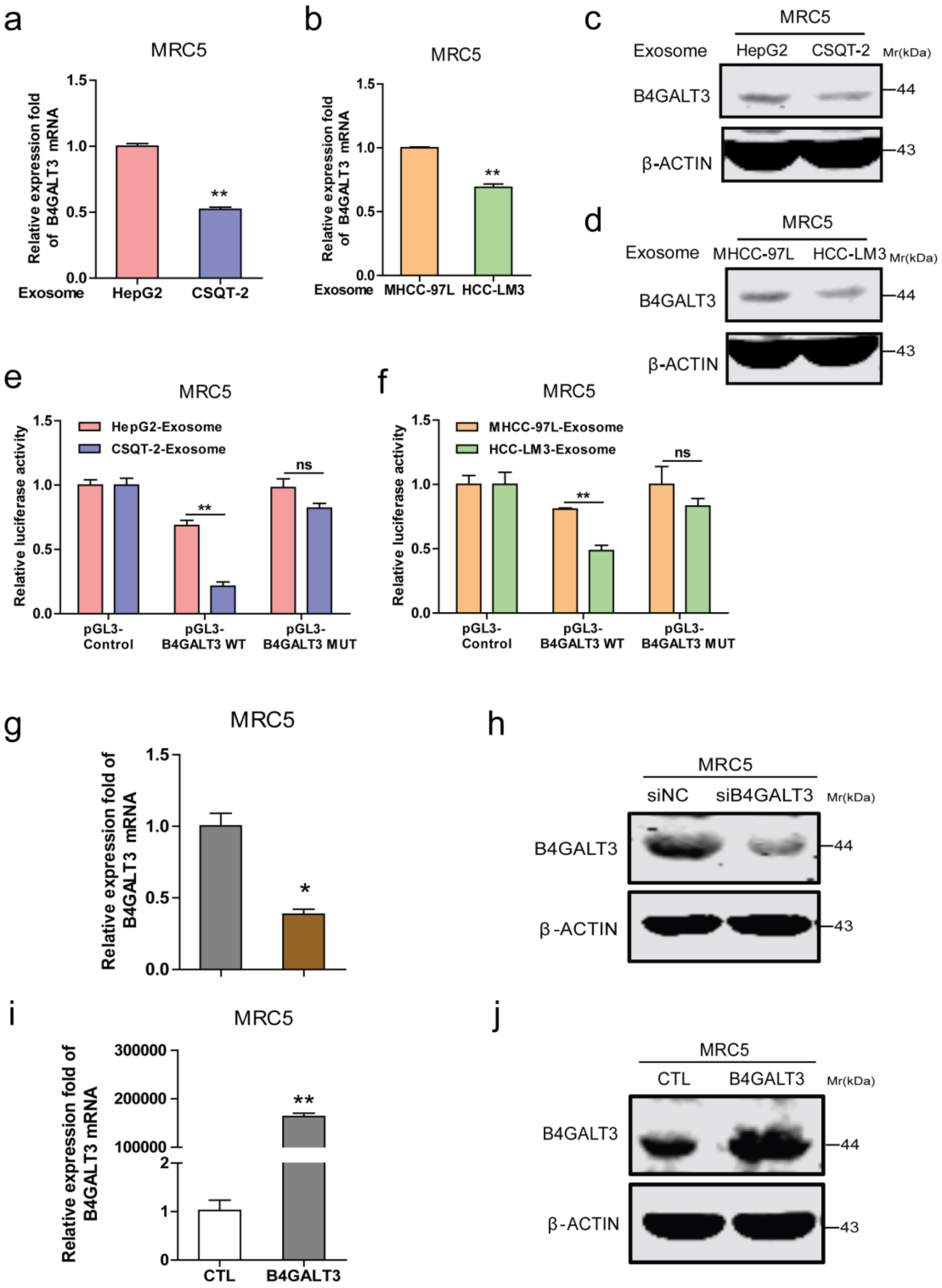


**Supplementary Fig. 3** B4GALT3 is the downstream target of highly metastatic cells-derived exosomes in fibroblasts activation. **a**, **b** B4GALT3 mRNA level in MRC5 treated with different tumor exosomes was detected by qRT-PCR analysis. **c**, **d** Immunoblotting assays of B4GALT3 expression in MRC5 treated with different tumor exosomes. **e**, **f** Relative luciferase activity of B4GALT3 in MRC5 treated with different tumor exosomes. **g-j** qRT-PCR and immunoblotting assays of B4GALT3 in MRC5 with

indicated treatments. Results are shown as mean ± s.d. Student's *t* test was used to analyze the data. (*p<0.05; **p<0.01; ***p<0.001)

Supplementary Fig. 4

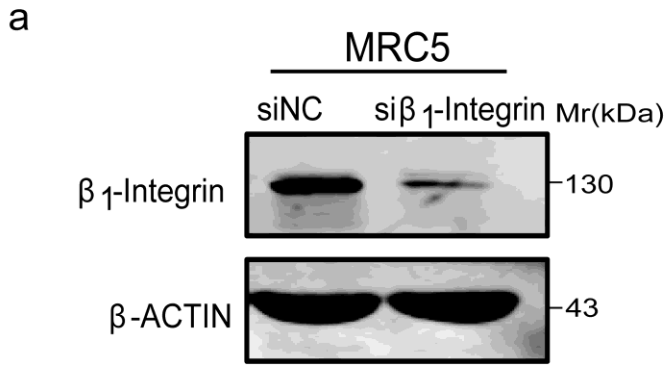


**Supplementary Fig. 4** The effect of β1-integrin knockdown in MRC5. **a** Immunoblotting assays of β1-integrin in MRC5 treated with siRNAs targeting β1-integrin or normal control.

Supplementary Fig. 5

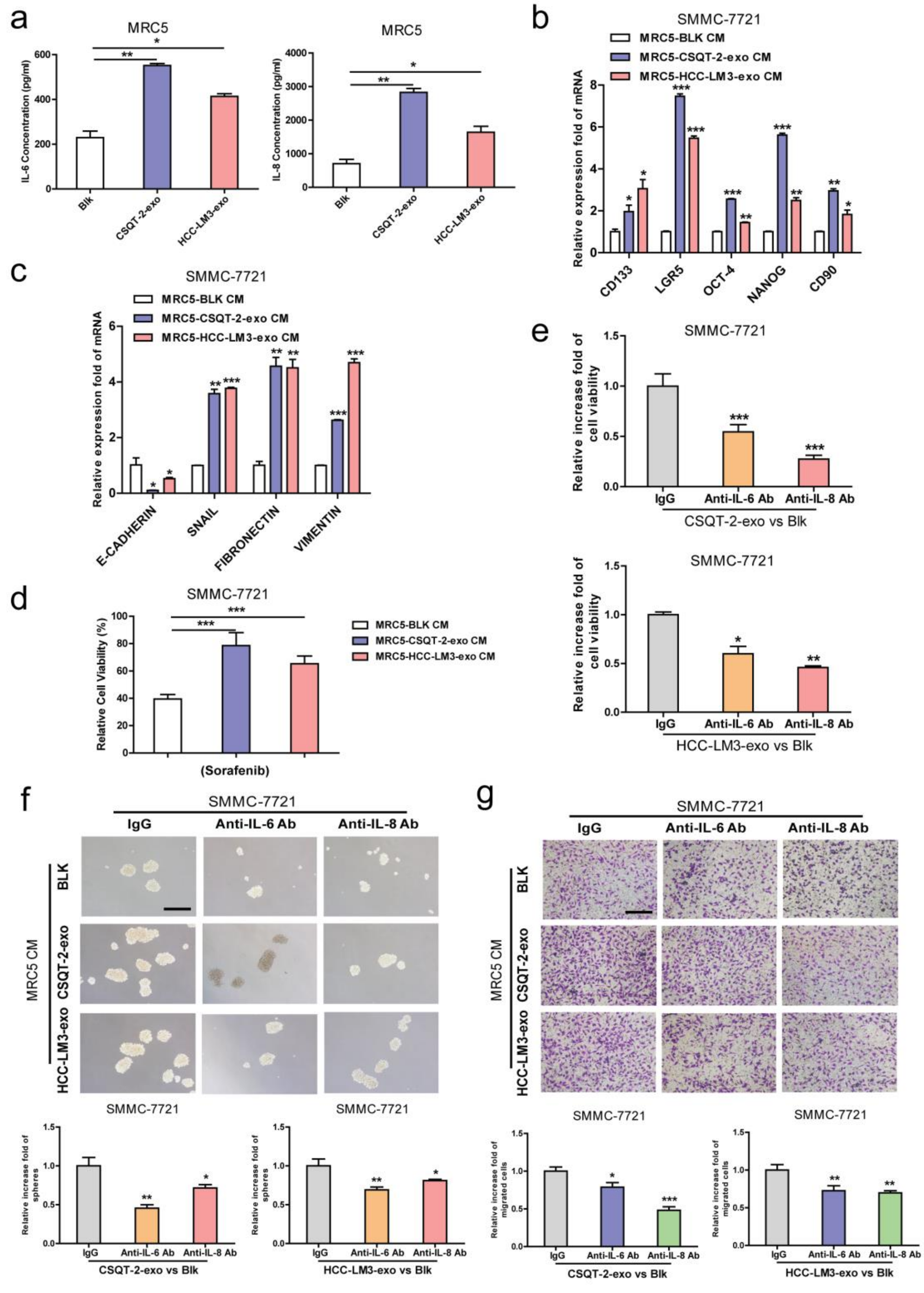


**Supplementary Fig. 5** Activated fibroblasts by highly metastatic cells-derived exosomes promote liver cancer progression. **a** IL-6 and IL-8 secretion from MRC5 treated with exosomes derived from highly metastatic HCC cell lines (CSQT-2 and HCC-LM3) or blank control. **b** qRT-PCR analysis of stemness-associated genes expression in SMMC-7721 with indicated treatments. **c** qRT-PCR analysis of EMT-associated genes expression in SMMC-7721 with indicated treatments. **d** Relative cell viabilities of SMMC-7721 treated

with indicated CM in presence of sorafenib. **e** Relative cell viabilities of SMMC-7721 treated with indicated CM containing anti-IL-6/ anti-IL-8 antibody or IgG control antibody in presence of sorafenib. **f** Spheroid formation ability of SMMC-7721 treated with indicated CM containing anti-IL-6/ anti-IL-8 antibody or IgG control antibody. Representative images were shown and spheroid were counted. Scale bar, 150 μm. **g** Migration assay of SMMC-7721 treated with indicated CM containing anti-IL-6/ anti-IL-8 antibody or IgG control antibody. Representative images were shown and migrated cells were counted. Scale bar, 150 μm. Each experiment was performed in triplicate and data are presented as mean ± s.d. Student's *t* test was used to analyze the data. (*p<0.05; **p<0.01; ***p<0.001)

Supplementary Fig. 6

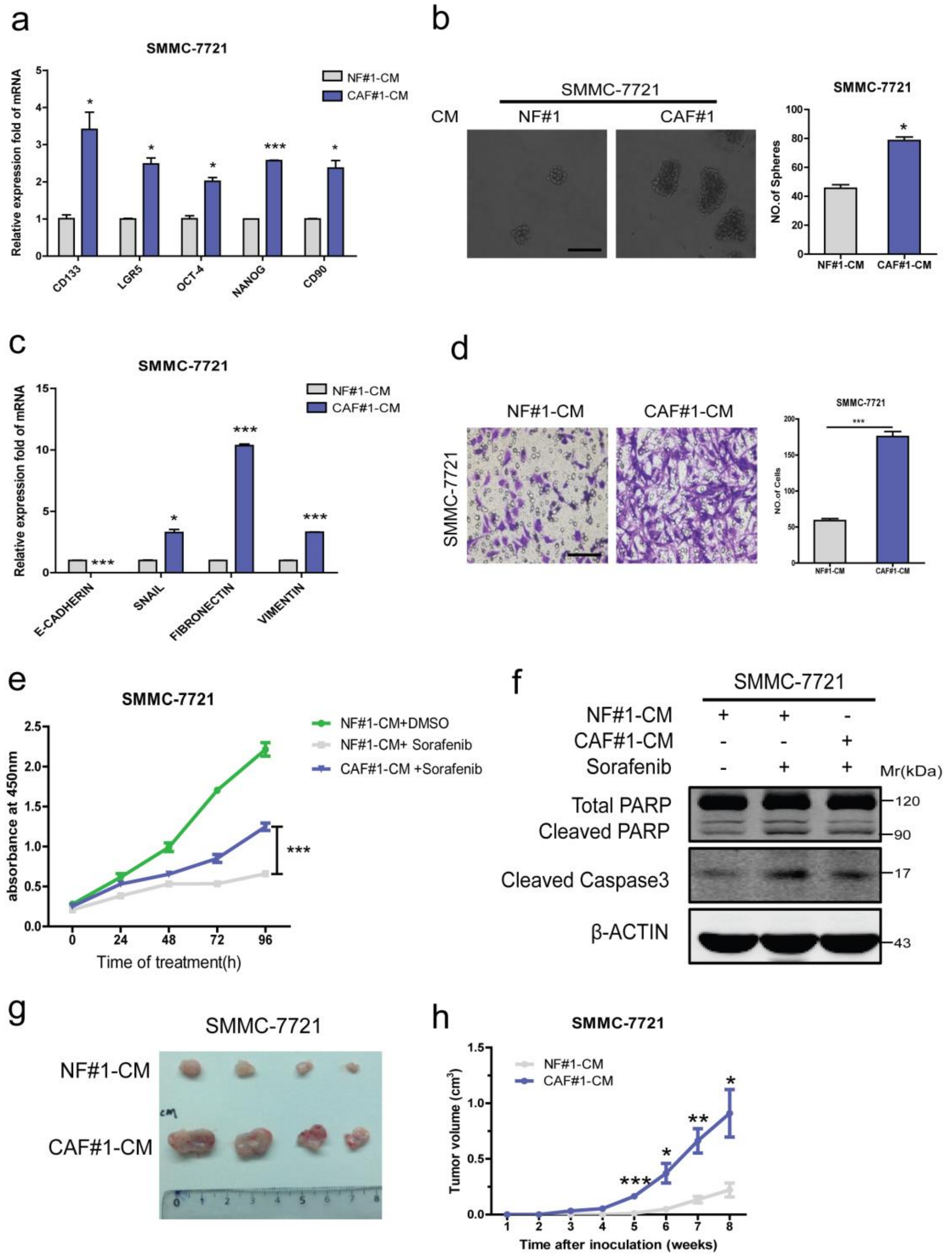


**Supplementary Fig. 6** Primary cancer-associated fibroblasts (CAFs) contribute to liver cancer progression. **a**, **c** qRT-PCR analysis of indicated genes expression in SMMC-7721 with indicated treatments. **b**, **d** Spheroid formation and migration assay of SMMC-7721 with indicated treatments. Representative images were represented and spheroid or migrated cells were counted. Scale bar, 150 μm. **e** CCK8 assay of SMMC-7721 with indicated treatments. **f** Western blotting assay of indicated proteins in SMMC-7721 with

indicated treatments. **g**, **h** Xenograft assays of SMMC-7721 with indicated treatments were carried out on nude mice. Representative tumors and tumors growth curves were shown. Data are presented as mean ± s.d. Student's *t* test was used to analyze the data. (*p<0.05; **p<0.01; ***p<0.001)

Supplementary Fig. 7

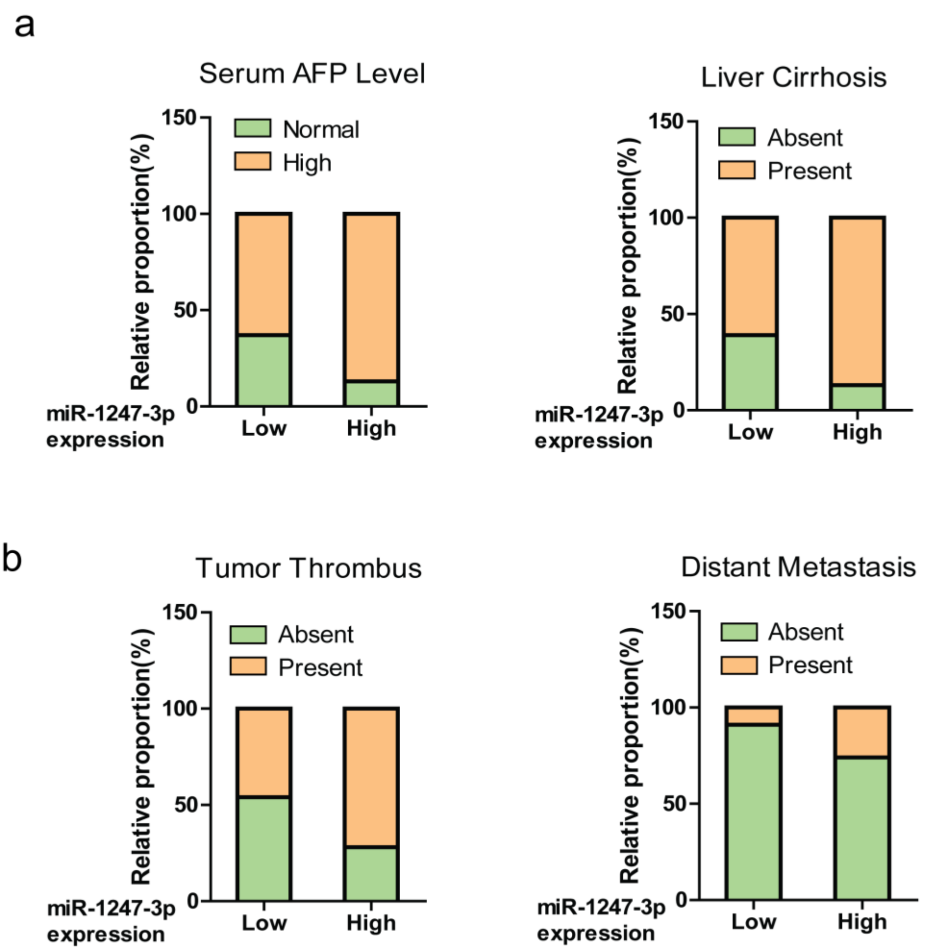


**Supplementary Fig. 7** The correlation of miR-1247-3p expression with clinicopathological features in HCC patients. **a**, **b** High miR-1247-3p expression was correlated with increased AFP level, liver cirrhosis, tumor thrombus, and distant metastasis in 85 HCC patients.

## Supplementary Fig. 8

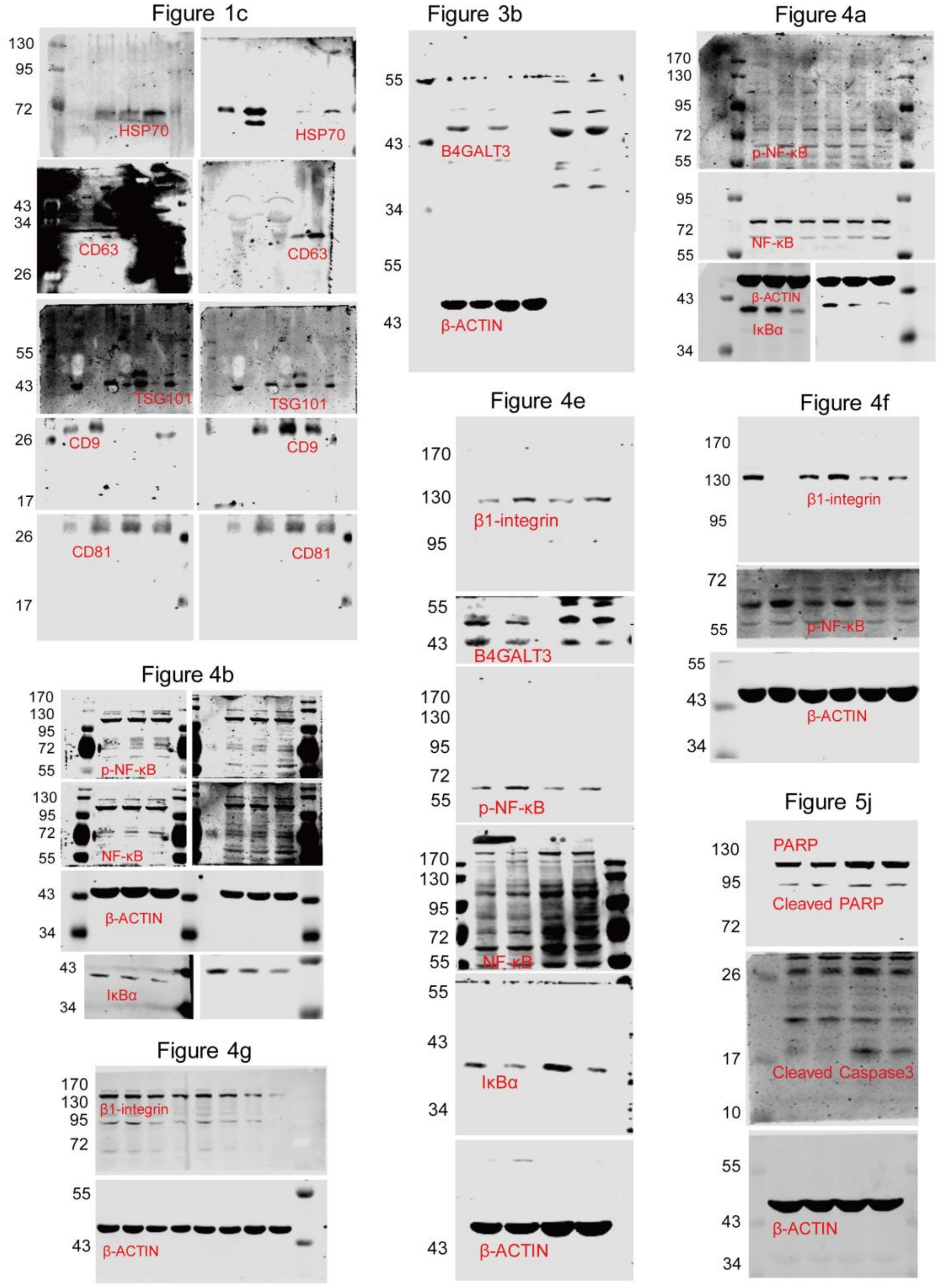


**Supplementary Fig. 8** The uncropped scans of western blots from the main figures.

**Supplementary Table 1** Summary of Clinicopathologic Variables.

| Variables | miR-1247-3p expression (number of cases) | | P-value |
|---|---|---|---|
| | Low（n=46） | High（n=39） | |
| **Age** | | | |
| ＜50 | 27 | 23 | P＞0.05 |
| ≥50 | 19 | 16 | |
| **Gender** | | | |
| Male | 41 | 33 | P＞0.05 |
| Female | 5 | 6 | |
| **HBV infection** | | | |
| Yes | 42 | 34 | P＞0.05 |
| No | 4 | 5 | |
| **Tumor size** | | | |
| ＜5cm | 15 | 10 | P＞0.05 |
| ≥5cm | 31 | 29 | |
| **Tumor number** | | | |
| Single | 27 | 15 | P＞0.05 |
| Multiple | 19 | 24 | |
| **AFP** | | | |
| ＜20ng/ml | 17 | 5 | **P＜0.05** |
| ≥20ng/ml | 29 | 34 | |
| **Tumor differentiation** | | | |
| Ⅰ-Ⅱ | 13 | 11 | P＞0.05 |
| Ⅲ-Ⅳ | 33 | 28 | |
| **Liver cirrhosis** | | | |
| Yes | 28 | 34 | **P＜0.05** |
| No | 18 | 5 | |
| **Tumor thrombus** | | | |
| Present | 21 | 28 | **P＜0.05** |
| Absent | 25 | 11 | |
| **Distant metastasis** | | | |
| Yes | 4 | 10 | **P＜0.05** |
| No | 42 | 29 | |

**Supplementary Table 2** Sequences of primers and siRNAs.

| Gene | Sequences |
|---|---|
| *IL-1β* | 5'-ATGATGGCTTATTACAGTGGCAA-3'<br>5'-GTCGGAGATTCGTAGCTGGA-3' |
| *IL-6* | 5'-ACTCACCTCTTCAGAACGAATTG-3'<br>5'-CCATCTTTGGAAGGTTCAGGTTG-3' |

| | |
|---|---|
| *IL-8* | 5'-TTTTGCCAAGGAGTGCTAAAGA-3'<br>5'-AACCCTCTGCACCCAGTTTTC-3' |
| *RAB27A* | 5'-GCTTTGGGAGACTCTGGTGTA-3'<br>5'-TCAATGCCCACTGTTGTGATAAA-3' |
| *B4GALT3* | 5'-CGAGATCAGGGACCGACATTT-3'<br>5'-GATCGTTCTGGACAGTAGGGC-3' |
| *CD133* | 5'-GCCACCGCTCTAGATACTGC-3'<br>5'-TGTTGTGATGGGCTTGTCAT-3' |
| *LGR5* | 5'-CTCCCAGGTCTGGTGTGTTG-3'<br>5'-GAGGTCTAGGTAGGAGGTGAAG-3' |
| *NANOG* | 5'-CATGAGTGTGGATCCAGCTTG-3'<br>5'-CCTGAATAAGCAGATCCATGG-3' |
| *OCT-4* | 5'-AGTGAGAGGCAACCTGGAGA-3'<br>5'-ACACTCGGACCACATCCTTC-3' |
| *CD90* | 5'-CTAGTGGACCAGAGCCTTCG-3'<br>5'-GCACGTGCTTCTTTGTCTCA-3' |
| *E-CADHERIN* | 5'-TGCCCAGAAAATGAAAAAGG-3'<br>5'-GTGTATGTGGCAATGCGTTC-3' |
| *SNAIL* | 5'-CCTCCCTGTCAGATGAGGAC-3'<br>5'-CCAGGCTGAGGTATTCCTTG-3' |
| *FIBRONECTIN* | 5'-CAGTGGGAGACCTCGAGAAG-3'<br>5'-TCCCTCGGAACATCAGAAAC-3' |
| *VIMENTIN* | 5'-GAGAACTTTGCCGTTGAAGC-3'<br>5'-GCTTCCTGTAGGTGGCAATC-3' |
| *18S* | 5'-CGGCTACCACATCCAAGGAA-3'<br>5'-GCTGGAATTACCGCGGCT-3' |
| si*RAB27A* | 5'-GGAGAGGUUUCGUAGCUUA-3' |
| si*B4GALT3* | 5'-CUACUGUCCAGAACGAUCUdTdT-3'<br>5'-AGAUCGUUCUGGACAGUAGdTdT-3' |
| si*ITGB1* | 5'-CAGCCCAUUUAGCUACAAAdTdT-3'<br>5'-UUUGUAGCUAAAUGGGCUGdTdT-3' |

**Supplementary Table 3** Sequences of miRNA mimics.

| Mimics | Sequences |
|---|---|
| hsa-miR-365a-5p | 5'-AGGGACUUUUGGGGGCAGAUGUG-3'<br>5'-CACAUCUGCCCCCAAAAGUCCCU-3' |
| hsa-miR-4494 | 5'-CCAGACUGUGGCUGACCAGAGG-3'<br>5'-CCUCUGGUCAGCCACAGUCUGG-3' |
| hsa-miR-4513 | 5'-AGACUGACGGCUGGAGGCCCAU-3'<br>5'-AUGGGCCUCCAGCCGUCAGUCU-3' |
| hsa-miR-1247-3p | 5'-CCCCGGGAACGUCGAGACUGGAGC-3'<br>5'-GCUCCAGUCUCGACGUUCCCGGGG-3' |
| hsa-miR-4688 | 5'-UAGGGGCAGCAGAGGACCUGGG-3'<br>5'-CCCAGGUCCUCUGCUGCCCCUA-3' |
| hsa-miR-4749-5p | 5'-UGCGGGGACAGGCCAGGGCAUC-3'<br>5'-GAUGCCCUGGCCUGUCCCCGCA-3' |
| hsa-miR-4758-3p | 5'-UGCCCCACCUGCUGACCACCCUC-3'<br>5'-GAGGGUGGUCAGCAGGUGGGGCA-3' |
| hsa-miR-513a-3p | 5'-UAAAUUUCACCUUUCUGAGAAGG-3'<br>5'-CCUUCUCAGAAAGGUGAAAUUUA-3' |
| hsa-miR-513b-3p | 5'-AAAUGUCACCUUUUUGAGAGGA-3'<br>5'-UCCUCUCAAAAAGGUGACAUUU-3' |
| hsa-miR-5684 | 5'-AACUCUAGCCUGAGCAACAG-3'<br>5'-CUGUUGCUCAGGCUAGAGUU-3' |
| hsa-miR-597-3p | 5'-UGGUUCUCUUGUGGCUCAAGCGU-3'<br>5'-ACGCUUGAGCCACAAGAGAACCA-3' |
| hsa-miR-659-3p | 5'-CUUGGUUCAGGGAGGGUCCCCA-3'<br>5'-UGGGGACCCUCCCUGAACCAAG-3' |
| hsa-miR-6730-5p | 5'-AGAAAGGUGGAGGGGUUGUCAGA-3'<br>5'-UCUGACAACCCCUCCACCUUUCU-3' |
| hsa-miR-6754-5p | 5'-CCAGGGAGGCUGGUUUGGAGGA-3'<br>5'-UCCUCCAAACCAGCCUCCCUGG-3' |
| hsa-miR-6772-5p | 5'-UGGGUGUAGGCUGGAGCUGAGG-3'<br>5'-CCUCAGCUCCAGCCUACACCCA-3' |

| | |
|---|---|
| hsa-miR-6775-3p | 5'-AGGCCCUGUCCUCUGCCCCAG-3'<br>5'-CUGGGGCAGAGGACAGGGCCU-3' |
| hsa-miR-6796-5p | 5'-UUGUGGGGUUGGAGAGCUGGCUG-3'<br>5'-CAGCCAGCUCUCCAACCCCACAA-3' |
| hsa-miR-6801-3p | 5'-ACCCCUGCCACUCACUGGCC-3'<br>5'-GGCCAGUGAGUGGCAGGGGU-3' |
| hsa-miR-6834-3p | 5'-UAUGUCCCAUCCCUCCAUCA-3'<br>5'-UGAUGGAGGGAUGGGACAUA-3' |
| hsa-miR-6890-5p | 5'-CAUGGGGUAGGGCAGAGUAGG-3'<br>5'-CCUACUCUGCCCUACCCCAUG-3' |
| hsa-miR-711 | 5'-GGGACCCAGGGAGAGACGUAAG-3'<br>5'-CUUACGUCUCUCCCUGGGUCCC-3' |
| hsa-miR-1247-3p inhibitor | 5'-GCUCCAGUCUCGACGUUCCCGGGG-3' |
